# Ventricular TLR4 Levels Abrogate TLR2-Mediated Adverse Cardiac Remodeling upon Pressure Overload in Mice

**DOI:** 10.3390/ijms222111823

**Published:** 2021-10-30

**Authors:** Elise L. Kessler, Jiong-Wei Wang, Bart Kok, Maike A. Brans, Angelique Nederlof, Leonie van Stuijvenberg, Chenyuan Huang, Aryan Vink, Fatih Arslan, Igor R. Efimov, Carolyn S. P. Lam, Marc A. Vos, Dominique P. V. de Kleijn, Magda S. C. Fontes, Toon A. B. van Veen

**Affiliations:** 1 Department of Medical Physiology, Division of Heart & Lungs, University Medical Center Utrecht, Utrecht University, 3584CM Utrecht, The Netherlands; gjmkok@hotmail.com (B.K.); M.A.D.Brans@umcutrecht.nl (M.A.B.); angelique.nederlof@gmail.com (A.N.); L.vanStuijvenberg@umcutrecht.nl (L.v.S.); M.A.Vos@umcutrecht.nl (M.A.V.); magda_fontes@hotmail.com (M.S.C.F.); A.A.B.vanVeen@umcutrecht.nl (T.A.B.v.V.); 2 Laboratory Experimental Cardiology, Division of Heart & Lungs, University Medical Center Utrecht, Utrecht University, 3508GA Utrecht, The Netherlands; Fatih.nl@gmail.com; 3 Department of Surgery, Yong Loo Lin School of Medicine, National University of Singapore, 10 Medical Dr, Singapore 117597, Singapore; surwang@nus.edu.sg (J.-W.W.); huangchenyuan@u.nus.edu (C.H.); 4 Cardiovascular Research Institute, National University Heart Centre Singapore, Singapore 117599, Singapore; 5 Department of Physiology, Yong Loo Lin School of Medicine, National University of Singapore, 10 Medical Dr, Singapore 117597, Singapore; 6 Nanomedicine Translational Research Programme, Centre for NanoMedicine, Yong Loo Lin School of Medicine, National University of Singapore, 10 Medical Dr, Singapore 117597, Singapore; 7 Department of Pathology, University Medical Center Utrecht, 3508GA Utrecht, The Netherlands; a.vink@umcutrecht.nl; 8 Department of Cardiology, Division of Heart & Lungs, University Medical Center Utrecht, Utrecht University, 3508GA Utrecht, The Netherlands; 9 Department of Biomedical Engineering, George Washington University, Washington, DC 20052, USA; efimov@email.gwu.edu; 10 National Heart Centre Singapore and Duke-National University of Singapore, 5 Hospital Dr, Singapore 169609, Singapore; carolyn.lam@duke-nus.edu.sg; 11 UMC Groningen, Hanzeplein 1, 9713GZ Groningen, The Netherlands; 12 Department of Vascular Surgery, The Netherlands & Netherlands Heart Institute, University Medical Center Utrecht, Utrecht University, 3508GA Utrecht, The Netherlands; dkleijn@umcutrecht.nl; 13 The Netherlands Heart Institute, Moreelsepark 1, 3511EP Utrecht, The Netherlands

**Keywords:** toll-like receptors, TLR2, TLR4, heart failure, pressure overload, inflammation

## Abstract

Involvement of the Toll-like receptor 4 (TLR4) in maladaptive cardiac remodeling and heart failure (HF) upon pressure overload has been studied extensively, but less is known about the role of TLR2. Interplay and redundancy of TLR4 with TLR2 have been reported in other organs but were not investigated during cardiac dysfunction. We explored whether TLR2 deficiency leads to less adverse cardiac remodeling upon chronic pressure overload and whether TLR2 and TLR4 additively contribute to this. We subjected 35 male C57BL/6J mice (wildtype (WT) or TLR2 knockout (KO)) to sham or transverse aortic constriction (TAC) surgery. After 12 weeks, echocardiography and electrocardiography were performed, and hearts were extracted for molecular and histological analysis. TLR2 deficiency (*n* = 14) was confirmed in all KO mice by PCR and resulted in less hypertrophy (heart weight to tibia length ratio (HW/TL), smaller cross-sectional cardiomyocyte area and decreased brain natriuretic peptide (BNP) mRNA expression, *p* < 0.05), increased contractility (QRS and QTc, *p* < 0.05), and less inflammation (e.g., interleukins 6 and 1β, *p* < 0.05) after TAC compared to WT animals (*n* = 11). Even though TLR2 KO TAC animals presented with lower levels of ventricular TLR4 mRNA than WT TAC animals (13.2 ± 0.8 vs. 16.6 ± 0.7 mg/mm, *p* < 0.01), TLR4 mRNA expression was increased in animals with the largest ventricular mass, highest hypertrophy, and lowest ejection fraction, leading to two distinct groups of TLR2 KO TAC animals with variations in cardiac remodeling. This variation, however, was not seen in WT TAC animals even though heart weight/tibia length correlated with expression of TLR4 in these animals (r = 0.078, *p* = 0.005). Our data suggest that TLR2 deficiency ameliorates adverse cardiac remodeling and that ventricular TLR2 and TLR4 additively contribute to adverse cardiac remodeling during chronic pressure overload. Therefore, both TLRs may be therapeutic targets to prevent or interfere in the underlying molecular processes.

## 1. Introduction

Hypertension due to chronic pressure overload is a major cardiovascular risk factor affecting a billion individuals worldwide [1,2]. Pressure overload leads to cardiac remodeling associated with cardiac stiffness and arrhythmias, eventually culminating in heart failure (HF) [1,3]. During adverse cardiac remodeling, activation of inflammatory pathways, such as the toll-like receptor (TLR) pathway, is a common observation [4,5]. TLRs are crucial for initiation of the innate immune system by recognizing damage- and pathogen-associated molecular patterns (DAMPs and PAMPs, respectively) [6].

Besides TLR4, which is the most abundant and well-studied TLR in the heart, TLR2 is the next most abundant cardiac TLR. Both are expressed in immune and cardiac non-immune cells (e.g., cardiomyocytes, fibroblasts, and vascular endothelial cells) [5,7], but knowledge about TLR2′s involvement in adverse cardiac remodeling is scarce. Upon ligand binding and activation of the TLR2 and TLR4 receptor, transcription factors such as the nuclear factor kappa-light-chain-enhancer of activated B cells (NF-κB) are translocated to the nucleus. This induces expression of inflammatory cytokines and chemokines (e.g., interleukin-1beta (IL-1β), IL-6 and tumor necrosis factor-alpha (TNF-α)), leading to an invasion of pro-inflammatory cells [8].

Elevated TLR2 levels have already been associated with ischemia reperfusion injury, adverse remodeling after myocardial infarction (MI), contractile dysfunction, atherosclerosis, and HF after TAC; and TLR2 as well as TLR4 are, e.g., increased in the circulation during chronic HF, after acute MI and are predictive for atrial fibrillation after MI [9,10,11,12,13]. TLR4 expression levels are associated with left ventricular (LV) dysfunction in patients undergoing coronary artery bypass surgery [14] and are known to stimulate adverse cardiac remodeling in various animal models. Upregulation of TLR4 in rat cardiomyocytes worsens cardiac function after long-term MI [15]. We and others previously showed that TLR4 deficiency in mice results in a lower degree of cardiac hypertrophy after MI or pressure overload induced by transverse aortic constriction (TAC) [16,17,18].

As both TLR4 and TLR2 can activate the same downstream targets, such as NF-κB, ablation of NF-κB protects against ventricular dilatation and fibrosis after MI and preserves LV function in mice [19]. Moreover, cardiac-specific inhibition of NF-κB protects against ischemia reperfusion injury by increasing Ca^2+^ re-uptake into the sarcoplasmic reticulum, suggesting a role for both receptors in maladaptive remodeling [20]. In general, TLRs can form homo- and heterodimers, enhancing or inhibiting receptor signaling, e.g., TLR2 and TLR4 have been suggested to form heterodimers in the brain and arteries; however, thus far, this has not been reported in cardiac muscle [6,21,22]. 

In this study, we hypothesized that TLR2 deficiency by knockout (KO) will lead to an amelioration of adverse cardiac remodeling in mice subjected to chronic pressure overload and that TLR2 and TLR4 additively contribute to this process. Therefore, both TLRs could serve as modifiable targets to prevent or interfere in the underlying molecular mechanism.

## 2. Results

In order to investigate whether ventricular TLR2 deficiency decreases adverse cardiac remodeling during pressure overload, a TLR2 KO mouse model was subjected to either sham or TAC surgery. The consistency of TAC surgeries was confirmed by measuring pressure gradients at the aortic constriction sites 2, 6, and 12 weeks after surgery (Table 1 and Supplementary Figure S1a). TLR2 deficiency (*n* = 14) was confirmed in all KO mice by PCR (Supplementary Figure S1b). Mortality caused by TAC surgery was not significantly different between WT and TLR2 KO mice (36% in TLR2 KO TAC vs. 51% in WT TAC animals (*p* = 0.33 using a log-rank Mantel–Cox test), there was no TAC-associated mortality among sham mice). 

### 2.1. TLR2 Deficiency Improves Cardiac Function and Contractility

**Figure 1:**
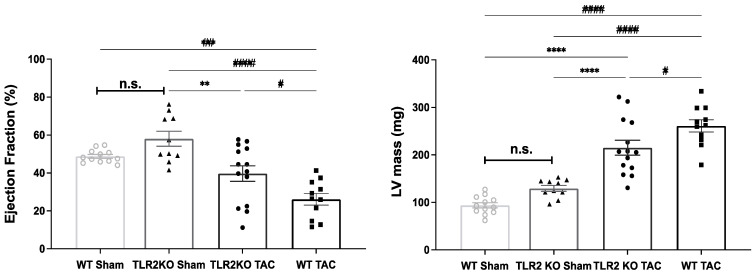
Echocardiographic parameters show no significant differences between WT sham and TLR2 KO sham group. All mice were obtained from Jackson Laboratory (C57BL/6J WT and TLR2 KO mice). WT sham (gray circles) and TLR2 KO sham (triangles) groups are comparable in their ejection fraction (%) and left ventricular mass (LVmass) (mg) measured by echocardiography after 8 and 12 weeks, respectively. Statistics were performed using a one-way ANOVA followed by Tukey’s multiple comparisons test. Black circles represent TLR2 KO TAC animals and black squares WT TAC animals. n.s. = not significant. ** *p* < 0.01, **** *p* < 0.0001; # *p* < 0.005, ## *p* < 0.01, #### *p* < 0.0001.

At 12 weeks after surgery, echocardiography and electrocardiography (ECG) were performed. Echocardiographic data of a WT sham group (as described in Material and Methods) indicated that the WT sham and TLR2 KO sham groups were not significantly different in their ejection fraction (EF) and left ventricular mass (LVmass) as shown in Figure 1 and Table 1. In addition to that, WT TAC animals showed a reduced contractility and cardiac function with a significantly decreased EF, fractional shortening (FS), and cardiac output (CO) compared to sham animals (Table 1). Interestingly, TLR2 KO TAC animals did not show these significant reductions compared to sham animals. Only stroke volume (SV) was significantly decreased in TLR2 KO TAC compared to TLR2 KO sham. In fact, almost all other echocardiographic data measured in WT TAC mice differed significantly from the sham group, while this appeared to be far less pronounced in the TLR2 KO TAC animals when compared to the respective TLR2 KO sham animals (Table 1).

ECG analysis upon TAC showed prolonged QRS intervals in WT TAC mice (12.7 ± 0.4 ms), which were significantly shorter in TLR2 KO TAC mice (11.4 ± 0.3 ms, Table 1). Furthermore, increased QTc intervals in the WT TAC group were significantly shortened in TLR2 KO after TAC (57.0 ± 2.3 ms in WT TAC vs. 51.1 ± 1.9 ms in TLR2 KO TAC, Table 1). 

### 2.2. TLR2 Deficiency Attenuates Inflammation

**Figure 2:**
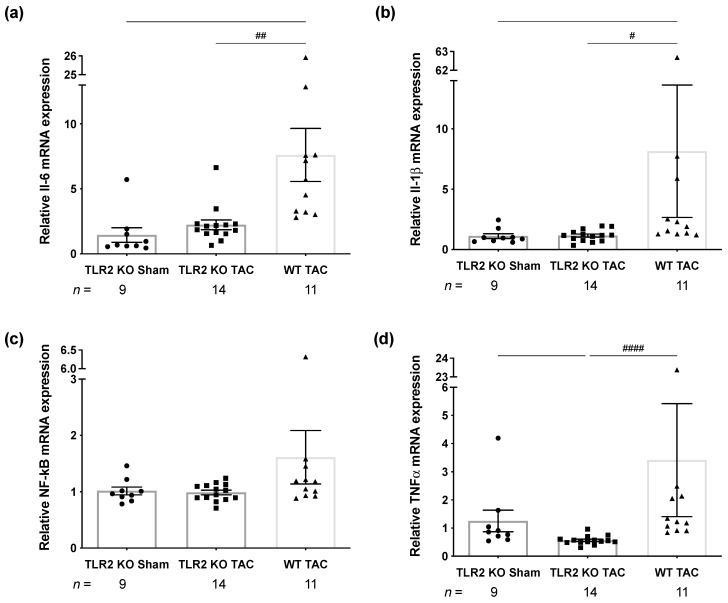
Inflammatory parameters in TLR2 KO and WT mice 12 weeks after sham or TAC surgery. (**a**) Relative mRNA expression of interleukin-6 (IL-6) by TaqMan RT-qPCR. (**b**) Relative mRNA expression of IL-1β assessed by TaqMan RT-qPCR. (**c**) Relative mRNA expression of nuclear factor kappa-light-chain-enhancer of activated B cells (NF-κB) by TaqMan RT-qPCR. (**d**) Relative mRNA expression of tumor necrosis factor-alpha (TNF-α) by TaqMan RT-qPCR. Circles represent TLR2 KO TAC animals, squares TLR2 KO TAC animals and triangles WT TAC animals. n indicates number of mice per group. For IL-6, a one-way ANOVA followed by Tukey’s test was used, and for the rest, a Kruskal–Wallis followed by Dunn’s multiple comparison test was used: # *p <* 0.05, ## *p <* 0.01, #### *p <* 0.0001.

To investigate the consequences of TLR2 deficiency on inflammation, the downstream targets of TLR4 and TLR2 activation were examined. Cardiac mRNA levels of the cytokines IL-6, IL-1β, and TNF-α were significantly decreased in the TLR2 KO TAC, when compared to the WT TAC group (Figure 2a,b,d). Transcription factor NF-κB was, despite of a tendency suggesting the highest expression level in WT TAC, not statistically different between all groups (Figure 2c). 

### 2.3. TLR2 Deficiency in Mice Ameliorates Hypertrophic Remodeling and Level of TLR4

**Figure 3:**
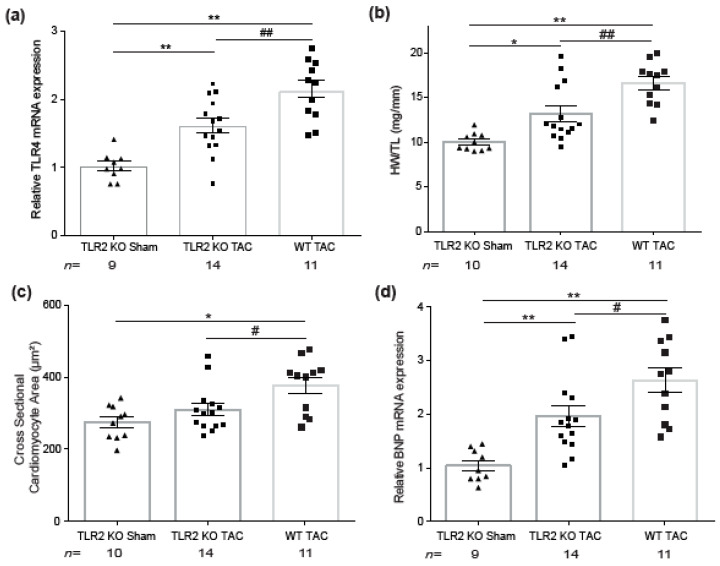
TLR4 and hypertrophic parameters in TLR2 KO and WT mice 12 weeks after sham or TAC surgery. (**a**) Relative mRNA expression of TLR4 assessed by TaqMan RT-qPCR. (**b**) Heart weight to tibia length ratio (HW/TL, mg/mm). (**c**) Cross-sectional cardiomyocyte area (μm^2^) assessed by fluorescent dystrophin labeling. (**d**) Ventricular mRNA expression of brain natriuretic peptide (BNP) assessed by TaqMan RT-qPCR. n indicates the number of mice per group. For TLR4, HW/TL, and BNP, a one-way ANOVA followed by Tukey’s test was used, and for the cross-sectional cardiomyocyte area, a one-way ANOVA followed by Tukey’s test on the log was used: * *p <* 0.05, ** *p <* 0.01, # *p <* 0.05 and ## *p <* 0.01.

Upon TAC, TLR4 mRNA levels appeared significantly lower in TLR2 KO mice (*p* = 0.005) compared to WT mice (Figure 3a). As we did not include a WT sham group, we afterwards compared mRNA expression of TLR2 and TLR4 between 8-week-old WT sham and TLR2 KO sham animals (Supplementary Figure S2), where depletion of TLR2 led to significant decrease of TLR2 (*p* = 0.004) as well as TLR4 (*p* = 0.005). In TLR2 KO TAC mice compared to WT TAC mice, this reduction of TLR4 levels was associated with a reduced degree of hypertrophy. HW/TL ratios were significantly lower in TLR2 KO TAC mice compared to WT TAC mice (13.2 ± 0.8 mg/mm versus 16.6 ± 0.7 mg/mm, respectively) and cardiomyocytes were smaller (309 ± 17 µm^2^ versus 376 ± 23 µm^2^, respectively) in TLR2 KO mice after TAC (Figure 3b,c and Table 2). As for consistency, mRNA levels of the hypertrophic marker brain natriuretic peptide (BNP) were lower in TLR2 KO mice compared to that in WT mice after TAC (Figure 3d). 

### 2.4. Increased TLR2 and TLR4 mRNA Gene Expression in Mice Subjected to Chronic Pressure Overload 

**Figure 4:**
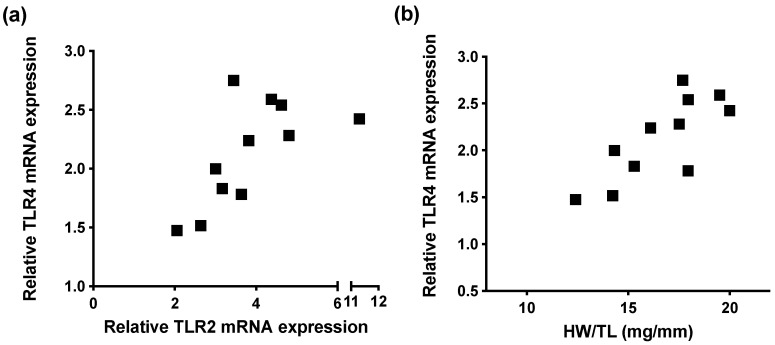
TLR4 and hypertrophy in mice upon chronic pressure overload. (**a**) Correlation between mRNA expression of TLR2 and TLR4 (r = 0.75; *p* = 0.01) assessed by TaqMan RT-qPCR in WT TAC mice in wildtype (WT) mice 12 weeks after transverse aortic constriction (TAC) surgery. (**b**) Correlation between heart weight to tibia length ratio (HW/TL, mg/mm) and ventricular mRNA expression of TLR4 (r = 0.78; *p* = 0.005). Correlations were analyzed using Pearson’s correlation.

In WT mice subjected to pressure overload (pressure gradient 61.7 ± 4.4 mmHg after 12 weeks of TAC), high levels of TLR2 correlated with high levels of TLR4 (r = 0.75; *p* = 0.01), suggesting interplay between the two TLRs (Figure 4a). In turn, high levels of TLR4 mRNA correlated with high levels of hypertrophy depicted as heart weight/tibia length ratios (HW/TL) in Figure 4b (r = 0.78; *p* = 0.005). 

### 2.5. High TLR4 Levels in TLR2 KO Animals Predict Adverse Cardiac Remodeling after TAC

**Figure 5:**
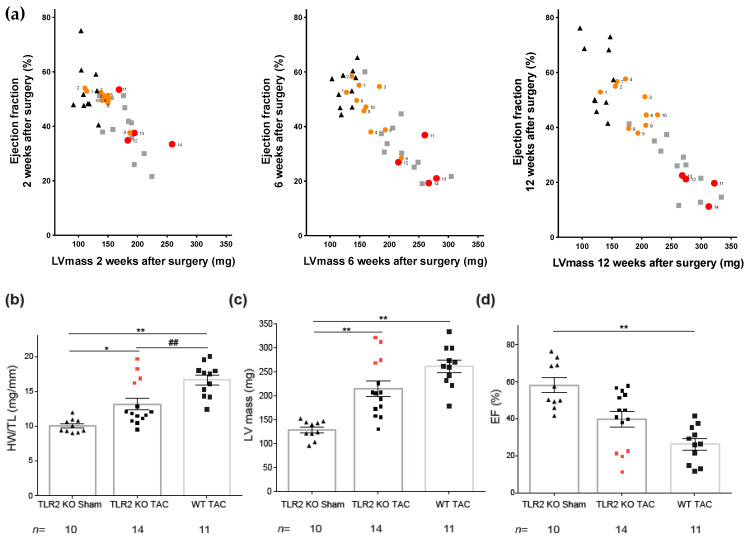
Mice with highest levels of TLR4 had the worst cardiac function. (**a**) Correlations between left ventricular mass (LV mass, mg) and ejection fraction (EF; %) over time at 2 weeks (left panel), 6 weeks (middle panel), and 12 weeks (right panel) after surgery. Black triangles depict mice of the TLR2 KO sham group, orange and red circles depict mice from the TLR2 KO TAC group, and gray squares represent mice from the WT TAC group. Red circles indicate mice with highest TLR4 mRNA levels. Numbers indicate the individual mice in the WT-TAC group (**b**) Heart weight to tibia length ratio (HW/TL, mg/mm) of TLR2 KO sham, TLR2 KO TAC, and WT TAC mice 12 weeks after surgery. (**c**) Left ventricular mass (LV mass, mg) measured by echocardiography after 12 weeks of TAC or sham surgery. (**d**) EF (%) measured by echocardiography after 12 weeks of TAC or sham surgery. n indicates the number of mice per group. A one-way ANOVA followed by Tukey’s test was used: * *p <* 0.05, ** *p <* 0.01, and ## *p <* 0.01.

Unexpectedly, during the 12-week follow up after TAC, TLR2 KO mice appeared to display two distinct phenotypes in response to pressure overload. This distinction developed over time as can be appreciated from the correlation between EF and LVmass at 2, 6, and 12 weeks after surgery (Figure 5a, mice of TLR2 KO group individually labeled for tracking), where we indicated the 4 aberrant animals of the TLR2 KO TAC group in red circles and the other 10 in orange circles. The far majority within the TLR2 KO TAC group (10/14 mice depicted in orange circles) showed a significant attenuated adverse ventricular remodeling when compared to the WT TAC group (11 mice, gray squares). A minority (4/14 mice, red circles) showed adverse cardiac structural and functional remodeling comparable to that observed in the WT TAC group (Figure 5a–d). The increased levels of TLR4 seemed to correlate with the increased HW/TL and the decreased EF as can be seen in Supplementary Figure S3. 

## 3. Discussion

The TLR4 pathway has been associated with HF and is involved in adverse cardiac remodeling and hypertrophy [2,16,23]. Whether, and how, TLR2 is able to modulate the proposed maladaptive role of TLR4 in cardiac remodeling is unknown. In this current study, we showed that ventricular TLR4 levels were high in failing murine hearts during cardiac dysfunction and, indeed, demonstrated a possible correlation of ventricular TLR2 and TLR4 during pressure overload. To achieve this, we investigated the effect of TLR2 deficiency on ventricular TLR4 in mice upon chronic pressure overload. 

As described in literature, increased levels of TLR4 mRNA have been reported in the hearts of TLR2-deficient mice after TAC [16,24]. In our study, TAC animals with TLR2 deficiency showed reduced levels of hypertrophy, as cell size, HW/TL, and levels of the hypertrophic marker BNP were significantly reduced when compared to those of WT TAC animals. Furthermore, these mice presented with near-normal cardiac function (ECG and echocardiographic measurements) and inflammatory parameters comparable to TLR2 KO sham animals, while WT TAC animals demonstrated adverse cardiac remodeling. 

We, therefore, concluded that TLR2 deficiency upon TAC generally preserves cardiac function and attenuates adverse cardiac remodeling. However, this appeared not to be a uniform observation, as the attenuation of adverse cardiac remodeling in the individual TLR2-deficient mice seemed to be imbalanced by (or co-depend on) the existing level of TLR4: mice with the highest degree of cardiac remodeling and failure showed high mRNA levels of TLR4 (Figure 5b–d, Supplementary Figure S3), while TLR2 deficiency was confirmed in all KO mice (Supplementary Figure S1b). 

Both TLRs activate similar downstream targets, e.g., NF-κB. Interestingly, it has been shown that depletion of TLR4 in endothelial cells can decrease the expression of TLR2 via the NFκB pathway [25] and that TLR2 is one of the target genes of NF-κB in, e.g., macrophages and monocytes [26,27]. This suggests a crosstalk between the two TLRs even with reference to expression levels. We, however, suggest that the crosstalk vice versa can also be NFκB independent, as in our study, NF-κB did not show changes in mRNA (Figure 2c) and protein level (data not shown) in mice at our time points. In addition to that, Supplementary Figure S4 shows that in TLR2 KO animals, mRNA levels of TLR4 were independent of NF-κB levels (low in sham and higher in TAC). In WT TAC animals, on the contrary, animals with highest TLR4 and NF-κB also had highest mRNA levels of TLR2 (red). 

To speculate, these findings could potentially explain the discrepancies seen in previous studies on TLR2. Wang et al. showed decreased fibrosis after TLR2 deficiency compared to WT animals [11]. Although they did not report TLR4 levels, downstream targets of both TLR2 and TLR4 (e.g., IL-1β, IL-6, and TNF-α) were decreased, suggesting that both pathways were inhibited [11]. Bualeong et al., however, reported that TLR2 deficiency could not attenuate the development of hypertrophy upon TAC, and their KO mice had significantly increased levels of TLR4 [28], which is in contradiction with our findings but could explain their increase in adverse cardiac remodeling. Spurthi et al. even showed that TLR2 KO increased hypertrophy, fibrosis, and apoptosis in aged mice compared to WT, even without a cardiac procedure [24]. Even though they did not observe increased TLR4 expression, they showed an increase in the transcription factor forkhead box protein O1 (Fox01), which has been shown to have TLR4 as a gene target in, e.g., macrophages, suggestive of increased TLR4 levels [29]. This suggests that TLR2 and TLR4 mainly work together under pathophysiological conditions, such as pressure overload. 

Interestingly, in a doxorubicin-induced mouse model, blocking of TLR2, but not TLR4 resulted in attenuation of cardiac dysfunction and inflammation [30]. In general, high TLR4 levels are associated with cardiac hypertrophy and are increased in the myocardium of patients with advanced HF [31]. In our study, we see that increased expression of TLR4 and TLR2 in WT mice upon chronic pressure overload was associated with higher levels of hypertrophy, suggesting at least a synergistic mode of action between the two TLRs. TLR4s are known to form homodimers with each other, and TLR2 and TLR4 are suggested to form heterodimers in the brain and arteries, but this has not yet been reported in the heart [6,21,22]. Furthermore, interaction on the protein level between TLR4 and TLR2 has been suggested after LPS and high-mobility group box-1 stimulation, although double TLR2–TLR4 KO experiments were not performed [32,33]. The amount of redundancy and expression of endogenous ligands, but also heterodimerization and interaction between TLR4 and TLR2 on both protein and gene expression levels, could, therefore, influence the outcome of cardiac remodeling, and TLR2 deficiency might be partially mediated by TLR4. To unravel the full mechanisms that drive adverse cardiac remodeling, future in-depth in vitro and interventional studies need to be performed.

### Study Limitations

In this study, we did not initially include a WT sham group, as literature data have already excluded differences in cardiac performance between this group and TLR2 KO sham animals [24,25]. However, in historical data of the same mice, we checked for potential differences in echocardiographic measures (EF and LVmass), which were not significantly different (Figure 1). We also analyzed TLR4 and BNP mRNA in WT sham and TLR2 KO sham animals, where upon TLR2 KO, BNP levels were similar, but TLR4 mRNA levels were significantly decreased. Lower TLR4 levels in TLR2 KO sham animals might suggest dependency of TLR4 on TLR2, which could influence the response upon pressure overload. This might also partly explain the differences seen in TLR2 KO TAC animals since the worst performing TLR2 KO TAC animals presented with the higher levels of TLR4 (Supplementary Figure S3) and adds to the hypothesis that both TLRs are suitable modifiable targets for anti-inflammatory treatments. The strong tendency of separated phenotypes within the TLR2 TAC group that we displayed in Figure 5 suggests differences between the subgroups, though applying statistics was not suitable because of the difference in size of the subgroups (4 vs. 10). Regarding the inflammatory proteins, we tried several Western blots but failed to provide specific bands. Because of that, expression data could not be translated into protein data. 

## 4. Materials and Methods 

### 4.1. Experimental Design for Mice

The animal experiments conducted conformed with the Guide for the Care and Use of Laboratory Animals published by the National Institutes of Health guide for the care and use of Laboratory animals (NIH Publications no. 8023, revised 1978) with the consent of the Experimental Animal Ethics Committee of the University Utrecht, The Netherlands (project identification 104699-3). All mice were male to minimize sex and hormonal influences and gender-dependent bias. TLR2 KO mice were commercially available (C57BL/6J WT and TLR2 KO mice from Jackson Laboratory, Bar Harbor, ME, USA), and global KO of TLR2 was confirmed by polymerase chain reaction (PCR). Mice were housed under normal laboratory conditions (a 12 h light/dark cycle with controlled humidity and temperature and food and water ad libitum) and were randomly distributed amongst groups regarding age, littermates, housing in the same cage, date, and time of surgery. After acclimatization, 10–12-week-old mice were randomly chosen to undergo transverse aortic constriction (TAC) or sham surgery under inhaled isoflurane anesthesia (2% in O_2_), as previously described [34]. Furthermore, all mice received subcutaneous injections of carprofen (5 mg/kg) as perioperative care. All mice were followed for 12 weeks and were analyzed in the following three groups: TLR2 KO sham (*n* = 10), TLR2 KO TAC (*n* = 14), and WT TAC (*n* = 11). As described in literature and shown previously by our group, TLR2 KO sham animals show HW/TL, heart rate (HR), and echocardiographic parameters that were comparable to WT sham animals [28,35]. Based on these published observations and to comply with the ethical goals of reduction, refinement, and replacement on experimental animal usage, inclusion of an additional WT sham group was omitted in our study. However, to facilitate easier comparison, we included data from a WT sham group of eight weeks of age (male mice of same strain and commercial origin; *n* = 12, data from [35]), as shown in Figure 1. Finally, all experiments were performed in a blinded fashion.

### 4.2. Genotyping

To confirm global TLR2 KO, PCR was performed on DNA isolated from liver biopsies using TLR2-specific oIMR3091 wildtype (5′-ACGAGCA AGATCAACAGGAGA-3′), oIMR3041 common/heterozygous (5′-CTTCCTGAATTT GTCCAGTA-3′) and oIMR3043 mutant (5′-GGGCCAGCTCA TTCCTCC CAC-3′) oligonucleotides. PCR amplification was repeated 35 times with Taq DNA polymerase (GE Healthcare, Buckinghamshire, UK) under the following conditions: denaturation for 30 s at 94 °C, annealing for 30 s at 65 °C, and elongation for 30 s at 72 °C. PCR products (wildtype 499 bp and mutant 334 bp) were visualized by agarose gel electrophoresis.

### 4.3. Electrocardiography and Echocardiography

Twelve weeks after surgery, mice were anesthetized with 2% isoflurane in O_2_, and a three-lead ECG was recorded using PowerLab 4/30 and Dual Bio Amp (AD Instruments Ltd., Oxford, UK). A minimum of 150 complexes were averaged and analyzed with LabChart 7 Pro (AD Instruments Ltd.). QTc was calculated using the Bazett’s formula. In addition, at 2, 6, and 12 weeks, transthoracic echocardiography was performed with the Vevo 2100 System (VisualSonics Inc., Toronto, Canada) using a 22–55 MHz transducer (MS550D). To confirm appropriate constriction in all TAC operated animals, aortic peak velocity was measured by pulsed-wave Doppler with a 13–24 MHz transducer (MS250). Analysis was performed with the Vevo 2100 software (VisualSonics Inc.).

### 4.4. Immunohistochemistry

Twelve weeks after surgery, mice were sacrificed by aortic transection, heart weights were determined, and subsequently hearts were snap-frozen in liquid nitrogen. Four-chamber-view cryosections of 10 μm were generated. To assess cell size, immunolabeling was performed using a mouse monoclonal antibody against dystrophin (1:1500, Sigma-Aldrich, Saint Louis, MI, USA) as described previously [36]. Secondary labeling was achieved by fluorescein isothiocyanate (FITC) anti-mouse whole IgG antibody (1:250, Jackson ImmunoResearch Europe, Newmarket, UK). In total, 10–15 randomly selected cells in randomly chosen images of three non-consecutive heart sections were analyzed per mouse with an epifluorescence microscope (Nikon Eclipse 80i; Nikon Europe BV, Amstelveen, The Netherlands) using the NIS Elements BR 3.0 software (Nikon Instruments Europe B.V., Amsterdam, The Netherlands). Quantification was performed using the ImageJ 1.48v software (National Institutes of Health, Bethesda, Maryland, USA). Cell size was defined as cross-sectional cardiomyocyte area, and cells with a Feret ratio >1.25 were excluded [37]. 

### 4.5. Real-Time Quantitative PCR (RT-qPCR)

For RT-qPCR on ventricular murine tissue, TaqMan Gene Expression Assays (all from Applied Biosystems by Life Technologies Corp., Carlsbad, CA, USA) were used as described earlier [36]. As an internal control, the geometric mean of succinate dehydrogenase complex flavoprotein subunit A (SDHA), ribosomal protein lateral stalk subunit P1 (RPLP-1), and TATA-binding protein (TBP) mRNA was used. Relative mRNA expression levels were determined for BNP, IL-6, IL-1β, NF-κB, TNF-α, TLR4, and TLR2. Assay IDs are listed in Supplementary Table S1.

### 4.6. Statistics

Data are expressed as mean ± standard error of the mean (SEM). Statistical analysis on murine data was performed using appropriate parametric or non-parametric tests (one-way ANOVA followed by Tukey’s multiple comparisons test or Kruskal–Wallis followed by Dunn’s multiple comparison test, respectively). We performed outlier testing, but none of the mice presented structurally as outliers, and therefore, we decided not to exclude mice based on one outlier gene. The survival data were analyzed using the log-rank test. Correlations were analyzed using Pearson’s correlation. All analyses were performed with GraphPad Prism 6.0 or 9.0 (GraphPad Software Inc., La Jolla, CA, USA), or SPSS (IBM SPSS Statistics for Windows 20.0, Armonk, NY, USA). A value of *p <* 0.05 was considered statistically significant.

## 5. Conclusions

TLR4 is associated with adverse cardiac remodeling in humans and mice subjected to pressure overload. Upon chronic pressure overload, TLR2 deficiency significantly tempered cardiac hypertrophy and preserved cardiac function in the vast majority of mice. In our study, ventricular TLR4 levels were high in failing murine hearts. In addition, TLR2-deficient mice with the highest levels of TLR4 mRNA showed the highest degree of cardiac dysfunction. Our data suggest that TLR2 deficiency ameliorates adverse cardiac remodeling and that ventricular TLR2 and TLR4 additively contribute to adverse cardiac remodeling during chronic pressure overload. Therefore, both TLRs may be therapeutic targets to prevent or interfere in the underlying molecular processes.

## Figures and Tables

**Table 1:** Echocardiographic and electrocardiographic measurements of TLR2 KO sham, TLR2 KO TAC, and WT TAC mice 12 weeks after surgery and historical WT sham after 8 weeks. Values are mean ± SEM for TLR2 KO sham, TLR2 KO TAC, and WT TAC mice and mean ± SD for WT sham mice. QTc was calculated using Bazett’s formula. LV, left ventricle; LVAW,s and LVAW,d, end-systolic and end-diastolic LV anterior wall thickness, respectively; LVPW,s and LVPW,d, end-systolic and end-diastolic LV posterior wall thickness, respectively; LVID,s and LVID,d, LV internal diameter end-systole and end-diastole, respectively; LV Vol,s and LV Vol,d, end-systolic and end-diastolic LV volume, respectively; EF, ejection fraction; FS, fractional shortening; SV, stroke volume; CO, cardiac output. * *p* < 0.05 and ** *p* < 0.01 compared to TLR2 KO sham; # *p* < 0.05 and ## *p* < 0.01 compared to TLR2 KO TAC.

	Sham		TAC	
	TLR2 KO	WT	TLR2 KO	WT
*n*	10	12	14	11
**Echocardiography**				
Pressure gradient (mmHg)	3.5 ± 0.3		61.6 ± 3.6 **	61.7 ± 4.4 **
LVAW,s (mm)	1.3 ± 0.1		1.5 ± 0.0	1.4 ± 0.1
LVAW,d (mm)	0.9 ± 0.1		1.1 ± 0.0 **	1.2 ± 0.1 **
LVPW,s (mm)	1.1 ± 0.1		1.3 ± 0.1	1.3 ± 0.1
LVPW,d (mm)	0.8 ± 0.0		1.1 ± 0.1 **	1.1 ± 0.1 **
LVID,s (mm)	2.8 ± 0.2		3.6 ± 0.2 *	4.2 ± 0.2 **
LVID,d (mm)	4.1 ± 0.1		4.4 ± 0.1	4.8 ± 0.2 **
LV Vol,s (μL)	31.4 ± 4.0		56.5 ± 8.7	81.7 ± 9.2 **
LV Vol,d (μL)	72.6 ± 3.3		88.3 ± 7.4	108.1 ± 8.4 **
LV mass (mg)	129.6 ± 6.1	93.9 ± 19.1	215.2 ± 15.7 **	261.1 ± 12.8 **
EF (%)	58.0 ± 4.0	48.8 ± 3.4	39.7 ± 4.1	26.1 ± 3.1 **
FS (%)	30.8 ± 2.7		19.6 ± 2.2	12.2 ± 1.5 **
SV (μL)	41.2 ± 1.7		31.7 ± 2.1*	26.4 ± 2.3 **
CO (mL/min)	19.0 ± 1.2		16.2 ± 1.1	13.3 ± 1.1 **
**Electrocardiography**				
Heart rate (bpm)	427.8 ± 18.4		479.8 ± 14.9	480.2 ± 15.5
RR (ms)	142.4 ± 5.7		126.6 ± 3.9 *	126.3 ± 4.2
PR (ms)	42.7 ± 0.8		42.8 ± 1.4	42.8 ± 1.5
P (ms)	9.8 ± 0.2		10.3 ± 0.3	11.2 ± 0.5
QRS (ms)	10.6 ± 0.3		11.4 ± 0.3	12.7 ± 0.4 **#
QTc (ms)	42.2 ± 0.6		51.1 ± 1.9	57.0 ± 2.3 **##

**Table 2:** Tissue characteristics of TLR2 KO sham, TLR2 KO TAC and WT TAC mice 12 weeks after surgery. Values are mean ± SEM. TAC, transverse aortic constriction; KO, knockout; WT, wild type; n, number of animals; HW/TL, heart weight to tibia length ratio; LuW/TL, lungs weight to tibia length ratio; LiW/TL, liver weight to tibia length ratio; KW/TL, average kidney weight to tibia length ratio. * *p <* 0.05 and ** *p <* 0.01 compared to TLR2 KO sham; # *p <* 0.05 and ## *p <* 0.01 compared to TLR2 KO TAC.

	Sham	TAC	
	TLR2 KO	TLR2 KO	WT
*n*	10	14	11
Body weight (g)	35.5 ± 0.8	33.9 ± 0.6	33.0 ± 0.5 *
Heart weight (mg)	184.2 ± 5.6	242.5 ± 15.9	304.6 ± 13.0 **
Tibia length (mm)	18.4 ± 0.1	18.4 ± 0.0	18.3 ± 0.1
HW/TL (mg/mm)	10.0 ± 0.3	13.2 ± 0.8 *	16.6 ± 0.7 **##
LuW/TL (mg/mm)	9.7 ± 0.2	11.8 ± 1.4	13.7 ± 2.0
LiW/TL (mg/mm)	83.5 ± 2.5	73.6 ± 2.6	80.7 ± 3.6
KW/TL (mg/mm)	10.6 ± 0.3	8.9 ± 0.2 **	9.7 ± 0.3 *#

## Data Availability

The data presented in this study are available on request from the corresponding author.

## Supplementary Materials

Supplementary materials can be found at https://www.mdpi.com/article/10.3390/ijms222111823/s1.
