# Supplementary material for: Ventricular TLR4 Levels Abrogate TLR2-Mediated Adverse Cardiac Remodeling upon Pressure Overload in Mice
Source: Int J Mol Sci. 2021 Oct 30;22(21):11823. doi: 10.3390/ijms222111823 (PMC8583975; doi:10.3390/ijms222111823)
Supplement: Supplementary file 1 [file ijms-22-11823-s001.zip › ijms-1411294-SI.pdf]

Supplementary material IJMS Kessler et al.

(a)

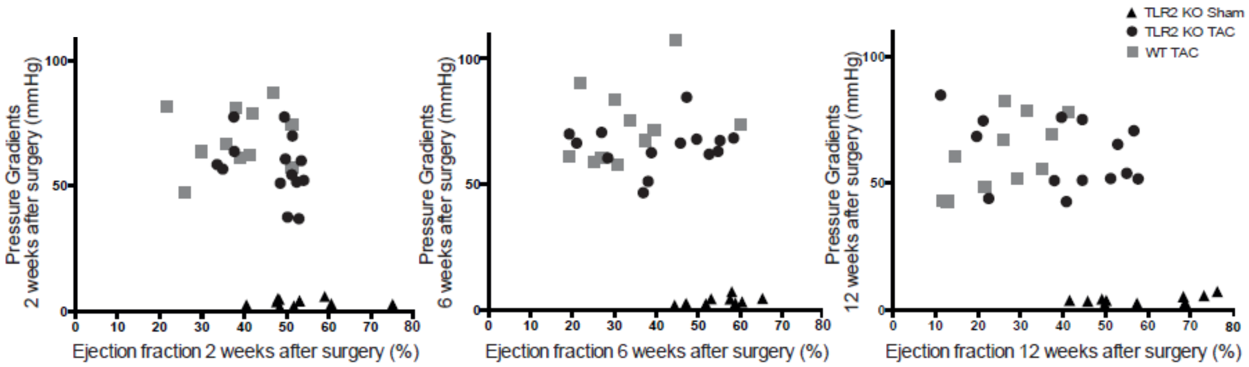

(b)

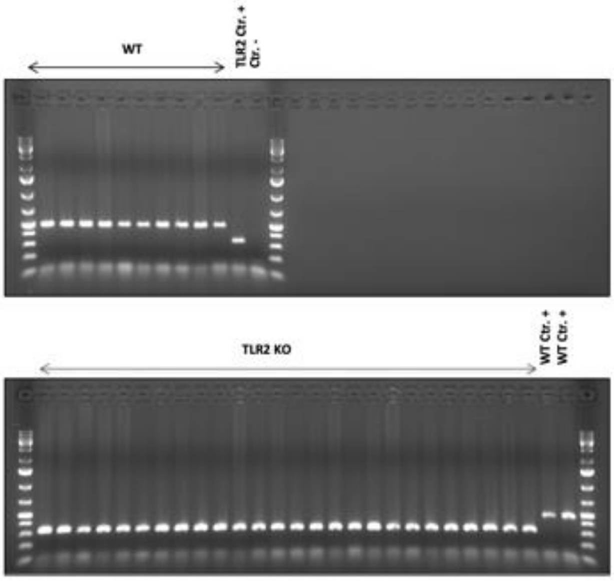

Figure S1. Aortic pressure gradients/ejection fractions and TLR2 expression in WT and TLR2 KO mice after Sham and TAC surgery.
(a) Ejection fraction (%) plotted against the aortic pressure gradients (mmHg) both measured by echocardiography after 2, 6, and 12 weeks of Sham and TAC surgery. **(b)** TLR2 expression measured by PCR confirms TLR2 deficiency in all KO mice (n=14) compared to WT mice and negative control.

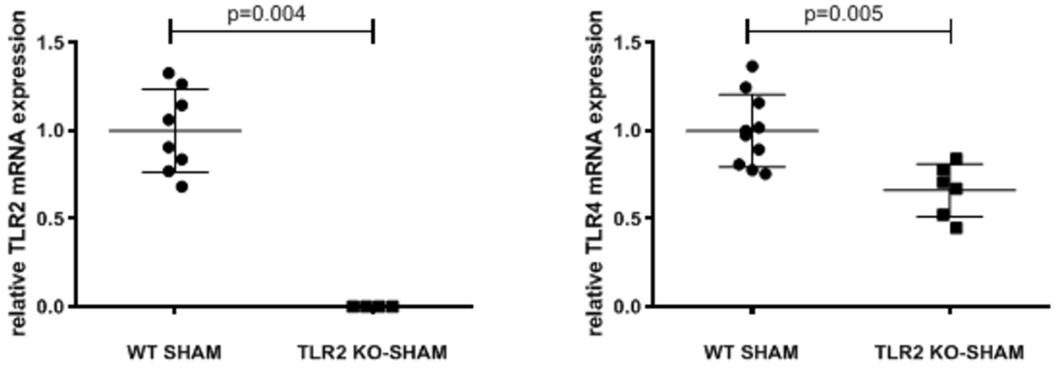

Figure S2. Ventricular mRNA expression of TLR2 (left) and TLR4 (right) assessed by RT-qPCR in wildtype (WT) Sham mice and TLR2 knockout (KO) Sham mice.

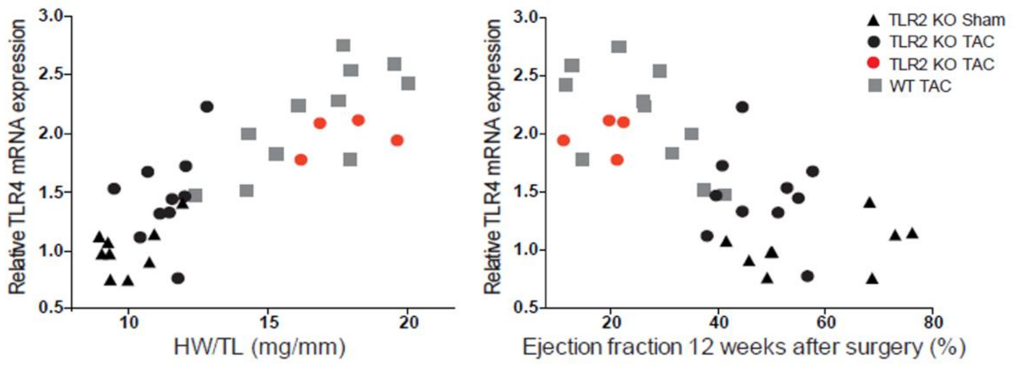

Figure S3. Correlation between mRNA expression of TLR4 assessed by RT-qPCR and heart weight to tibia length ratio (HW/TL, mg/mm; left graph) and ejection fraction (%); right graph.

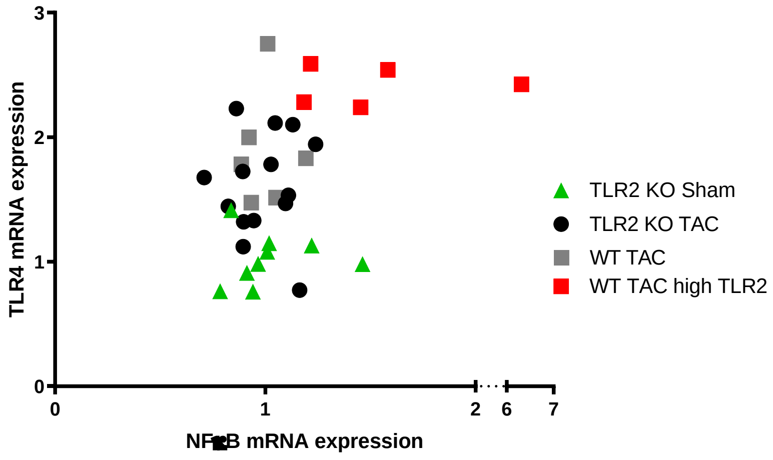

Figure S4. TLR4 and NFκB in mice upon chronic pressure overload. Correlation between ventricular mRNA expression of TLR4 and NFκB assessed by TaqMan RT-qPCR in TLR2 knockout (KO) Sham mice (green triangles), TLR2 KO transverse aortic constriction (TAC) mice (black circles), and wildtype (WT) TAC mice (gray squares indicate mice with low TLR2 levels and red squares mice with highest TLR2 levels).

Table S1. Characteristics of qPCR primers used in this study.
Protein, gene name and assay ID of all primers (all purchased from Applied Biosystems by Life Technologies Corp., Carlsbad, USA).

**Protein**	**Gene**	**Assay ID**
SDHA	*Sdha*	Mm01352366_m1
RPLP-1	*Rplp1*	Mm02601846_g1
TBP	*Tbp*	Mm00446971_m1
BNP	*Nppb*	Mm01255770_g1
IL-6	*Il6*	Mm00446190_m1
IL1-β	*Il1b*	Mm00434228_m1
NF-κB	*NF-κB*	Mm00477798_m1
TNF-α	*Tnf*	Mm00443258_m1
TLR4	*Tlr4*	Mm00445273_m1
TLR2	*Tlr2*	Mm00442346_m1
